# Alpha-Synuclein Is a Cellular Ferrireductase

**DOI:** 10.1371/journal.pone.0015814

**Published:** 2011-01-10

**Authors:** Paul Davies, Dima Moualla, David R. Brown

**Affiliations:** Department of Biology and Biochemistry, University of Bath, Bath, United Kingdom; National Institute on Aging Intramural Research Program, United States of America

## Abstract

α-synuclein (αS) is a cellular protein mostly known for the association of its aggregated forms with a variety of diseases that include Parkinson's disease and Dementia with Lewy Bodies. While the role of αS in disease is well documented there is currently no agreement on the physiological function of the normal isoform of the protein. Here we provide strong evidence that αS is a cellular ferrireductase, responsible for reducing iron (III) to bio available iron (II). The recombinant form of the protein has a V_Max_ of 2.72 nmols/min/mg and K_m_ 23 µM. This activity is also evident in lysates from neuronal cell lines overexpressing αS. This activity is dependent on copper bound to αS as a cofactor and NADH as an electron donor. Overexpression of α-synuclein by cells significantly increases the percentage of iron (II) in cells. The common disease mutations associated with increased susceptibility to PD show differences in activity or iron (II) levels. This discovery may well provide new therapeutic targets for PD and Lewy body dementias.

## Introduction

The 140 residue presynaptic protein α-synuclein (αS) has been implicated in the pathogenesis of several neurodegenerative disorders including Parkinson's disease (PD), Lewy body variant Alzheimer's disease and dementia with Lewy bodies (DLB) [1]. The symptoms of these conditions result from a loss of dopaminergic neurons, primarily from the pars compacta region of the substantia nigra. This then leads to a disruption of activity in basal ganglia that govern movement. Accompanied with these symptoms is an accumulation of ubiquitin bound αS within diseased cells [2]. The precise mechanism by which this is related to cell death remains unknown. Furthermore, a key issue in the fight against Lewy body disorders is that the physiological function of αS remains unknown, although it is thought to be related to membrane binding [3].

It has previously been suggested that αS is a copper binding protein[4]. Other studies have revealed a potentially significant association with iron [5], [6]. This factor may provide insight into its function as many proteins that bind redox active metals ions such as copper have a catalytic role. Previous investigations into the coordination of copper and iron binding to αS have failed to reach a consensus. Initial results showed αS to bind 5–10 molecules of copper [7], [8] while a later study showed αS to bind copper at two high affinity sites with more copper able to bind at other lower affinity sites [4]. A more recent study found two, independent, non-interacting Copper (II) binding sites in the N-terminus of αS [9].

The function of αS has often been suggested to be associated with dopamine metabolism and a variety of data indicate changes to dopamine synthesis, storage and transport processes[10]. In particular, a number of studies have suggested that overexpression of αS alters the activity of the key enzyme involved in dopamine synthesis, tyrosine hydroxylase[11], [12]. Dopamine synthesis in αS-knockout mice is reduced.[13]. Parkinson's disease shows changes to brain iron including the ratio of Fe(II) and Fe(III)[14]. Fe(II) is necessary for dopamine synthesis as it is a co-factor of tyrosine hydroxylase. Fe(III) may induce oxidative stress through interaction with neuromelanin [15]. Therefore, regulation of the ratio of Fe(II) to Fe(III) might be important for the survival of dopaminergic neurons. However, all these suggestions are inferential and no direct observation of a function has been made so far.

Our investigations of metal binding to αS lead us to investigate the potential of this interaction to endow function to the protein that might be relevant to the maintenance of Fe(II) levels in cells. Our findings demonstrate conclusively that αS is a ferrireductase.

## Results

### Metal Binding

**Figure 1:**
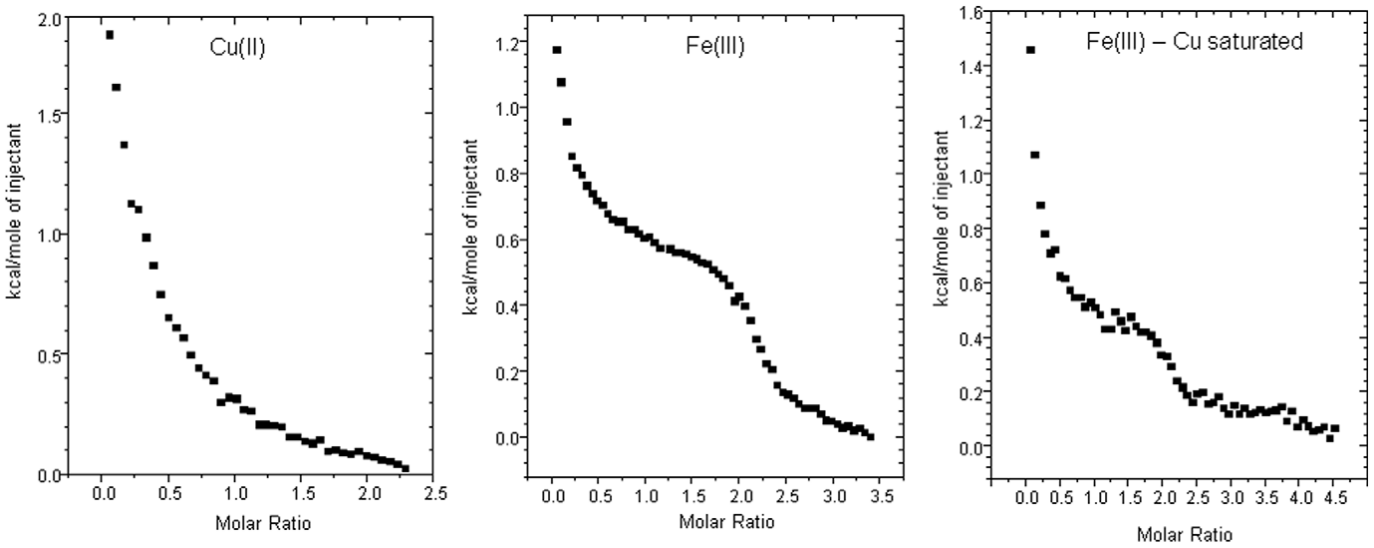
ITC analysis of copper and iron binding. ITC traces for titrations of Cu (II) or Fe (III) into 100 µM αS at pH 7, 25°C. An isotherm for the titration of Fe (III) into Cu (II) loaded protein is also shown (far right).

We first assessed the copper (II) and iron (III) binding to αS in order to gain insight into the significance of this association. Recombinant αS was cloned from human cell lines and expressed and purified by a method developed by our laboratory. Thermodynamic analysis using isothermal titration calorimetry (ITC) was used to assess the Cu(II) and Fe (III) binding dynamics of the protein. Figure 1 shows a representative isotherm for the delivery of glycine chelated copper (II) and unchelated iron (III) to αS. A titration of Fe (III) into Cu (II) loaded αS was also carried out. The isotherm for Copper (II) shows a net endothermic reaction as a result of the high enthalpy of copper disassociation from glycine. The overall effect of the glycine chelate on the isotherm was accounted for as previously described [16]. The fitting of a non-linear regression model (Microcal) to the data to the isotherm for Cu (II) revealed one binding site, with affinity of log 9.43 M^−1^. The titration of iron (III) into αS produced an exothermic reaction suggesting two binding sites (Figure 1). The affinity of Fe(III) for the protein was for log 5.02 M^−1^ both sites. A titration of iron (III) into protein that has been allowed to equilibrate with 2 equivalents of copper produced an exothermic reaction. The reaction indicated two sites with a affinity of log 4.50 M^−1^. Although the affinity at the sites was reduced the data indicated that αS can bind both copper and iron simultaneously. This then led us to hypothesise that copper loaded αS may be catalysing a redox process with the iron, perhaps reducing it to the ferrous form.

### Ferrireductase Activity in Recombinant Protein

**Figure 2:**
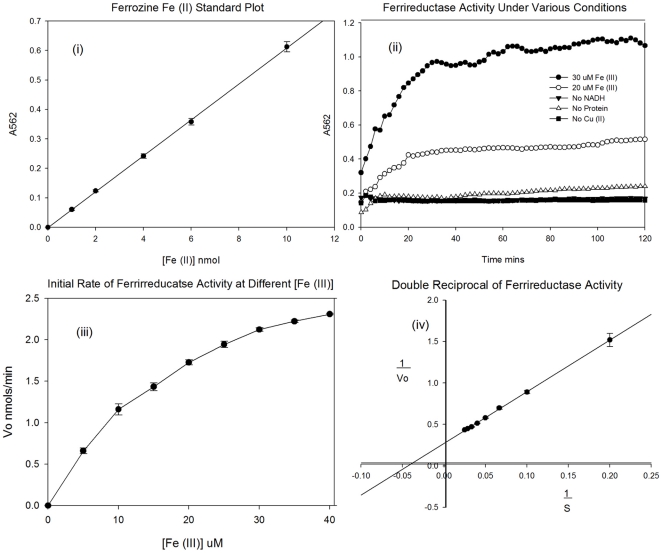
Kinetic plots of the ferrireductase activity of αS at 25°C. (i) Ferrozine calibration curve for standards of Fe (II) sulphate monitored at 562 nm. (ii) The change in absorbance for formation of ferrozine – Fe (II) complex at 562 nm for αS with either 20 mM or 30 mM Fe (III) and controls missing either protein, copper or NADH. (iii) Initial rates of activity dependent on substrate concentration. (iv) Double reciprocal plot for the calculation of Vmax and Km.

**Figure 3:**
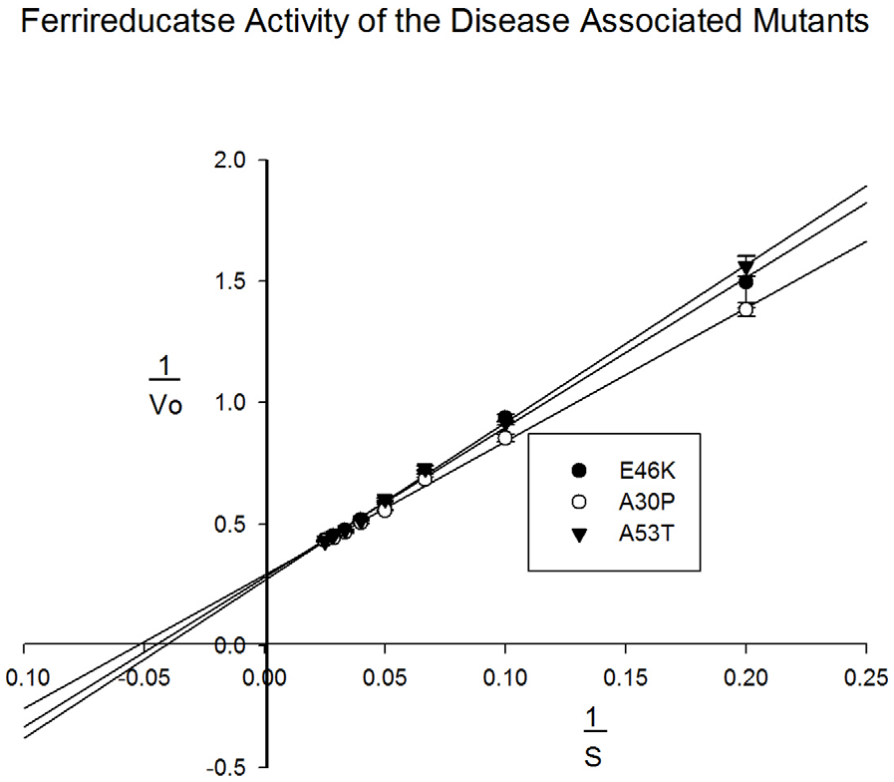
Comparison of the ferrireductase activity of the disease associated αS mutants.

In order to assess whether αS displayed ferrireductase activity, we used recombinant protein for an assessment of precise enzyme kinetics. The copper (II) bound protein was subjected to a ferrozine ((3-(2-pyridyl)-5,6- bis(phenyl sulfonic acid)-1,2,4-triazine), based assay to detect the reduction of iron (III) to iron (II), using NADH as an electron donor. Ferrozine forms a bis complex which is highly specific to the ferrous form of the ionic metal [17] and adsorbs light strongly at 562 nm. Figure 2(i) shows a linear plot of standard Fe^2+^ against absorbance for the determination of ferric iron concentrations. The ferric iron was added anaerobically as Fe (II) sulphate. For the assessment of αS ferrireductase activity Fe (III) was added as iron citrate. Controls in the absence of copper, protein or NADH were used to ensure any observed activity was due to the copper loaded protein. Figure 2(ii) shows the plot of the A562 against time for this experiment. For the copper loaded protein in the presence of NADH, concentrations of 20 µM and 30 µM Fe (III) citrate are shown. From the plot, it is clear that αS is able to catalyse the reduction of iron (III) to iron (II) in the presence of NADH and that this is dependent on the protein being loaded with copper (II). Increasing the concentration of substrate increases the rate of reaction in a predictable manner. Experiments were carried out with a range of substrate concentrations from 1 µM to 100 µM iron (III). Figure 2(iii) shows a plot of Vo against substrate concentration and figure 2(iv) shows a double reciprocal plot of this data. From these plots, the V_max_ and K_m_ can be calculated for the reaction (table 1). Certain gene mutations have been associated with an increased risk of PD [18] and as such these mutants were generated and tested to assess any affects on the ferrireductase activity of the protein. Figure 3 shows the double reciprocal plots for this data. From the plot, it is clear there is a small but significant affect on the activity of the protein. Table 1 summarises the key kinetic data obtained. We also analysed the homologue of αS, beta-synuclein (βS) but this protein had no activity in this assay (data not shown) suggesting the ferrireductase activity is specific to αS.

**Table 1:** Comparison of the kinetic parameters for the ferrireductase activity of αS and its disease associated mutants.

Mutant	Vmax (nmols/min/mg)	Km (µM)
Wildtype	2.72	23
E46K	2.52	23
A30P	2.37	19
A53T	2.62	25

Assays recorded at 562 nm and 25°C.

### αS Increases Cellular Ferriductase Activity and Fe(II) Levels

**Figure 4:**
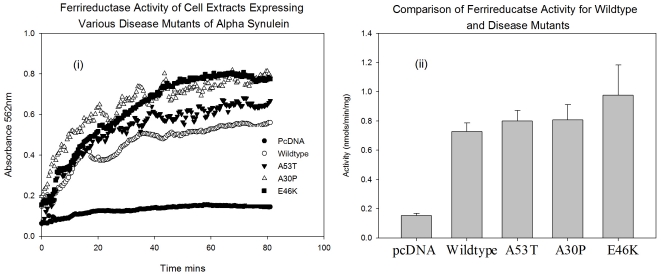
Cellular Ferrireductase Activity. Ferrireductase activity of lysates from neuronal cells normally or over expressing αS and its disease associated mutants. (i) The change in absorbance for formation of ferrozine – Fe (II) complex at 562 nm. (ii) A comparison of the initial rates.

With data obtained from recombinant protein, there is clearly a need to investigate whether cellular forms behave in a similar way. As such, we also transfected human neural cell lines with αS and its disease associated mutants to overexpress the protein in a cell culture based system. Lysates were then prepared and equal quantities of protein, assessed by Bradford assay and western blot, were assayed under identical conditions to those before. Figure 4(i) shows a plot of the A562 against time for the assay on the lysates from cells normally expressing αS and overexpressing the protein and its disease associated mutants. Figure 4(ii) shows the calculated initial rates form the experiment. There is a significant increase in ferrireductase activity in all the conditions using lysates from cells overexpressing αS when compared to lysates from cells normally expressing the protein. There was also a significant increase in activity in the lysates from cells overexpressing the E46K mutant. This suggests that this disease mutant behaves differently to normal αS with respect to its enzymatic activity in cells.

**Figure 5:**
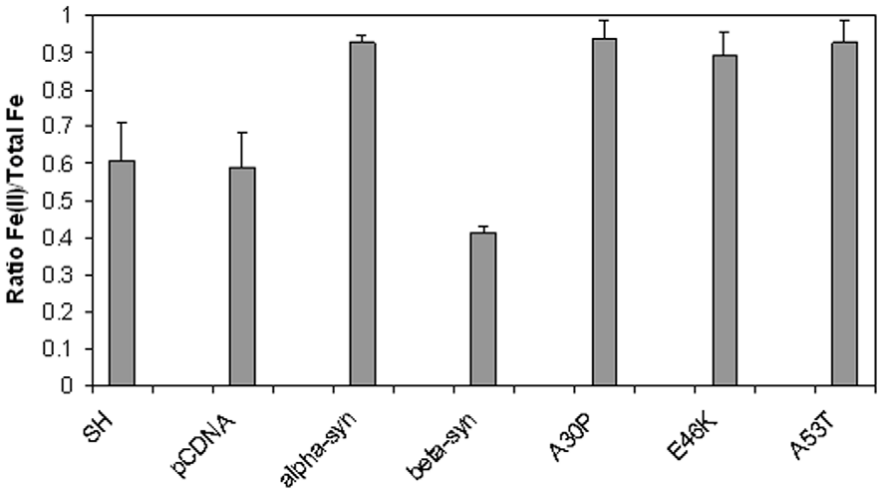
Iron Content of Cells. The levels of Fe(II) as compared to total cellular Fe (Fe(II)+Fe(III) in cell lines were assessed using a commercial assay. The cell lines were stably tranfected with either pCDNA, αS, the three αS mutants or βS. As a control the parental SH-SY5Y (SH) cell line was also analysed. Shown are the mean and standard error for three individual experiments with triplicate analyses.

In order to verify that increased cellular ferrireductase activity resulted in an increase in reduced iron in cells overexpressing αS or its mutants, the levels of ratio of Fe(II) total cellular Fe was determined for the stable cell lines using a commercial kit. The ratio of Fe(II) to total Fe is shown in Figure 5. As can be seen, overexpression of αS resulted in a significant increase in the proportion of Fe(II) present in the cells (Student's t test p<0.05). The three mutants of αS also showed significant increases but there was no significant difference between any of the mutants and wild-type αS. In contrast βS overexpression caused a small but significant decrease in the levels of Fe(II). This result indicates that the increase in Fe(II) is the result of overexpression of a protein with ferrireductase activity.

## Discussion

While the association of αS with a number of diseases has resulted in the expression “synucleinopathies” to describe them, the exact role of this protein in disease pathogenesis is poorly understood[19]. A potential avenue of investigation to identify the mechanism would be to identify the function of the protein and then establish how this activity is lost or altered in diseases such as Parkinson's disease. There have been a number of suggestions about the possible function of αS but none have been conclusively accepted[20]. The loss of dopaminergic neurons in Parkinson's disease has lead to some efforts in linking αS to some aspect of dopamine metabolism such as vesicular release[21]. The data presented here represents the first suggestion of an enzymatic activity for αS that could be of relevance for dopaminergic cells. This function also takes into account the metal binding capacity of the protein.

While analysis of copper binding to αS has been relatively extensive[4], [22], [23], [24], [25], [26], [27], there has been little consideration of iron in this regard[5]. Our findings not only show that αS can bind iron but demonstrate that this may be important for the normal activity of the protein. Differences in the affinity values report by us when compared to others in the literature probably reflect methodological differences. In particular we show a single copper binding site while others report two sites[4]. The use of chelators in our studies eliminates non-specific interaction and maintains copper in a more physiological form than those who use free copper. Also, MOPS is a buffer with significantly low interference with thermodynamic equilibria ensuring that our measurements are related only to metal-protein binding events. A single copper binding site has been reported by others using ITC and our data confirms their findings. In relation to iron our experiments were performed under conditions that resulted in little or no precipitation of Fe and the higher affinity we observe reflects this.

Our data is the first to show that αS can bind two different metals simultaneously. Cu(II) saturate αS was still able to bind Fe(III) with little change in affinity. This clearly indicates that the two metals occupy different sites on the protein. The majority of data suggest that copper binds to the N-terminus of αS while iron binds to the C-terminus. This finding would also suggest that there is a difference in the co-ordination of the metals. Some studies have suggested that αS can bind more copper than we have shown with ITC. Under these conditions copper binding might prevent iron binding at one or more sites. However, as previously suggest, replacement of iron by copper, and binding of copper at multiple sites on the protein might lead to aggregation of the protein. The potential of copper to enhance αS aggregation has been observed. In contrast, our suggestion is that both copper and iron can bind to the protein under normal physiological conditions and allow it catalyse the reduction of Fe(III) to Fe(II), indicative of a ferrireductase.

The data presented here suggests that αS is able to function as a cellular ferrireductase. This ties in well with recent evidence showing the protein associated with cell membranes [3]. Most cellular ferrireductases are found integral or closely associated to the bio-membrane [28]. Also, ferrireductases commonly utilise redox cycling of copper atoms to remove electrons from iron and thus reduce Fe(III) to Fe(II). The data we present shows kinetics that fit well with a simple enzyme following a Michaelis-Menten model. The activity requires an electron acceptor such as NADH and does not occur in the absence of copper. Significantly, the activity was observed with both recombinant protein and in cells overexpressing αS. Increased Fe (II) levels in the cell confirm that the ferrireductase assay measured a reduction process.

Our findings on αS activity are possibly important for understanding its cellular role. However, a key reason for studying αS is its role in diseases such as Parkinson's Disease. In Parkinson's disease the important change that occurs is the loss of dopamingeric neurons. When considering the potential role of a ferrireductase in dopaminergic cells then the generation of increased levels of Fe(II) may be important. A key enzyme in the dopamine pathway is tyrosine hydroxylase (TH) [29], an iron (II) dependent hydroxylase responsible for catalyzing the conversion of the amino acid L-tyrosine to dihydroxyphenylalanine (L-DOPA). Disruption of this pathway would occur if bio available Fe (II) was reduced as a result of a reduction of ferrireductase activity. Additionally, there is a build up of iron (III) in dopaminergic neurons in patients suffering from PD [30]. Clearly, a key pathological feature of Lewy body dementia is the aggregation of αS. It is reasonable to assume that aggregated protein would lose any enzymatic activity and hence have an affect on the ability of the cell to produce bio-available iron (II). Alternatively, increased expression of αS as occurs in Parkinson's disease may generate excess Fe(II) a metal that can catalyse the Fenton reaction and generate excess reactive oxygen species which may result in apoptosis. While these considerations are highly speculative, it does clearly indicate potential mechanisms where by altering ferrireductase activity could have a significant impact on the survival of dopaminergic neurons.

αS mutants identified from inherited forms of Parkinson's disease were also studied for their impact on Ferriductase activity and iron reduction. There was little evidence that these point mutations had any significant effect on this activity. This does not preclude the possibility that alterations in ferrireductase activity could be important for dopaminergic neuron survival. Partients with inherited forms of Parkinson's disease live a significant part of their lives without developing symptoms but have an earlier onset of symptoms than seen in sporadic. Therefore it is likely that these mutations have their impact in concert with age dependent changes. Thus a gross alteration of protein function is unlikely and has been confirmed by these findings.

In summary we have identified αS as a cellular ferrireductase. This provides the first finding of a function for this protein via direct observation rather than inference. Given the importance of maintaining Fe(II) levels in dopaminergic cells, this activity is likely to be of considerable importance to understanding the changes that occur in dopaminergic cells leading to their selective loss in Parkinson's disease.

## Methods

All reagents were purchased from Sigma unless otherwise stated.

### Recombinant Protein

αS and βS were produced as previously described [31] with the exception that the ammonium sulphate precipitation step was omitted. Additionally, purified protein was dialysed against MilliQ filtered water to a 1,000,000 times. Site directed mutagenesis was employed to create the disease associated mutants A30P, E46K and A53T of αS as previously described [16] using primers produced by MWG, Germany.

### Cell Culture

SH-SY5Y (human neuroblastoma) cells were cultured in 50% (v/v) Dulbecco's Modified Eagle Media and 50% (v/v) Ham's F-12 supplemented with 10% (v/v) fetal bovine serum (FBS), 100 U/ml penicillin, 100 µg/ml streptomycin and 2 mM glutamine. All the media and reagents were obtained from Biowhittaker Lonza. Growth conditions were maintained at 37°C and 5% CO_2_ in a humidified incubator.

### Transfections

Wildtype βS, αS and its disease associated mutants were subcloned into pcDNA plasmid transfected using FuGene 6 transfection reagent (Roche applied science). For stable transfections, 6-well plates were used and cells were plated at 50% confluency the day before the transfection. To perform the transfection 50 µl of serum free media was transferred into a micro-centrifuge tube; 3 µl of the FuGene 6 reagent was then added directly to this and mixed by gentle flicking. The mixture was incubated for 5 minutes at room temperature. For each cell line, 1 µg of DNA was then added to the mixture, mixed as before, and incubated at room temperature for 15 minutes. After this the transfection mixture was added to the cells, the 6-well plate swirled and returned to the incubator overnight. Cells were selected with 400 µg/ml G418 sulfate (Calbiochem) 24 hours after transfection, and maintained in 100 µg/ml to keep the plasmid in the cells.

### Isothermal Titration Calorimetry

ITC was carried out as previously described [16]. Various concentrations of protein were titrated with Cu (II): Glycine in a 1∶2 ratio to a final molar ratio of 1: 5. All solutions were buffered in 50 mM MOPS (3-(N-Morpholino)propanesulfonic acid) pH 7. For analysis of Fe(III) binding, αS was prepared at 250 µM and titrated with iron citrated 12.5 mM. Analysis of Fe(III) binding to copper saturated αS was also performed. In this case the αS was incubated with two molar equivalents of CuSO_4_ prior to ITC experiments.

### Ferrireductase Assay

Protein solutions of standardised cell lystates or to a final concentration of 100 µM recombinant protein were mixed with final concentrations of 100 µM ferrozine ((3-(2-pyridyl)-5,6- bis(phenyl sulfonic acid)-1,2,4-triazine), 50 µM NADH, 100 mM MOPS pH 7. Protein had been exposed to 4 equivalents of copper (II) sulphate and allowed to equilibrate. The reaction was started by the addition of iron (III) citrate to final concentrations between 1 µM and 100 µM to a final reaction volume of 100 µL. Reactions were carried out in 96 well plates and monitored at 562 nm in a BMG flurostar. All conditions wee repeated at least 8 times.

### Iron Assay

Levels of Fe(II) and total Fe were analysed in cell lines using the kit from Abcam and following the manufacturer's instructions. Cells were harvested from a confluent T75 for each analysis.
