# Correction: Alpha-Synuclein Is a Cellular Ferrireductase

**DOI:** 10.1371/annotation/900a5247-7d03-4686-a544-5f7f64c0aac5

**Published:** 2011-01-27

**Authors:** Paul Davies, Dima Moualla, David R. Brown

There was an error in the the second to last sentence of the Abstract: The key word "no" is missing. The correct sentence should be: "The common disease mutations associated with increased susceptibility to PD show no differences in activity or iron (II) levels."

